# Primary Ciliary Dyskinesia in Adult Bronchiectasis: Data from the German Bronchiectasis Registry PROGNOSIS

**DOI:** 10.1016/j.chest.2024.05.023

**Published:** 2024-06-15

**Authors:** Raphael Ewen, Isabell Pink, Sivagurunathan Sutharsan, Sven P. Aries, Achim Grünewaldt, Amelia Shoemark, Urte Sommerwerck, Ben O. Staar, Sabine Wege, Pontus Mertsch, Jessica Rademacher, Felix C. Ringshausen, PROGNOSIS Study Group, Borghild Grün, Bad Windsheim, Stefan Dargel, Katarina Ludwig, Andrés de Roux, Ralf Otto-Knapp, Hartmut Lode, Christian Gogoll, Meike Probst, Frank Herrmann, Axel Overlack, Stefan Pabst, Urte Sommerwerck, Köln; Harald Vehar, Stefan Blaas, Bernhard Schaaf, Martin Kolditz, Sivagurunathan Sutharsan, Essen; Peter Kardos, Achim Grünewaldt, Stephan Sorichter, Tobias Scholz, Marco Idzko, Moritz Mohadjer, Stephan Eisenmann, Sven P. Aries, Johannes Lauer-Hermfisse, Sabine Kampf, Felix C. Ringshausen, Sabine Wege, Felix Herth, Santiago Ewig, Christian Reinhardt, Stefan Andreas, Christian Schumann, Ingrid Bobis, Thomas Bahmer, Kiel; Rita Fey, Martin Jüch, Lostau; Axel T. Kempa, Erika Piirsoo, Benjamin Klapdor, Pontus Mertsch, Bernhard Schmidt, Holger Hein, Peter Haidl, Jorge Fernando Gamarra

**Affiliations:** a Department of Respiratory Medicine and Infectious Diseases, Hannover Medical School, Hannover, Germany; b Biomedical Research in End-stage and Obstructive Lung Disease Hannover, German Center for Lung Research, Hannover, Germany; c European Reference Network for Rare and Complex Lung Diseases, Frankfurt, Germany; d Department of Pulmonary Medicine, University Hospital Essen, Ruhrlandklinik, University Duisburg-Essen, Essen, Germany; e Elbpneumologie MVZ GmbH, Hamburg, Germany; f Department of Respiratory Medicine and Allergology, University Hospital, Goethe University Frankfurt, Frankfurt, Germany; g Respiratory Research Group, Division of Molecular and Clinical Medicine, University of Dundee, Dundee, Scotland; h PCD Diagnostic Service, Royal Brompton Hospital, London, England; i Department of Pneumology, Krankenhaus der Augustinerinnen Cologne, Cologne, Germany; j Department of Pneumology and Critical Care Medicine, Thoraxklinik at the University Hospital Heidelberg, Heidelberg, Germany; k Translational Lung Research Center Heidelberg, German Center for Lung Research, Heidelberg, Germany; l Department of Medicine V, University Hospital, LMU Munich, Comprehensive Pneumology Center Munich, Member of the German Center for Lung Research, Munich, Germany

**Keywords:** bronchiectasis, Kartagener syndrome, phenotype, primary ciliary dyskinesia, registries

## Abstract

**Background:**

Primary ciliary dyskinesia (PCD) is a rare genetic disorder caused by the malfunction of motile cilia and a specific etiology of adult bronchiectasis of unknown prevalence. A better understanding of the clinical phenotype of adults with PCD is needed to identify individuals for referral to diagnostic testing.

**Research Question:**

What is the frequency of PCD among adults with bronchiectasis; how do people with PCD differ from those with other etiologies; and which clinical characteristics are independently associated with PCD?

**Study Design and Methods:**

We investigated the proportion of PCD among the participants of the Prospective German Non-CF-Bronchiectasis Registry (PROGNOSIS) study; applied multiple imputation to account for missing data in 64 (FEV_1_), 58 (breathlessness), 26 (pulmonary exacerbations), and two patients (BMI), respectively; and identified predictive variables from baseline data using multivariate logistic regression analysis.

**Results:**

We consecutively recruited 1,000 patients from 38 centers across all levels of the German health care system. Overall, PCD was the fifth most common etiology of bronchiectasis in 87 patients (9%) after idiopathic, postinfective, COPD, and asthma. People with PCD showed a distinct clinical phenotype. In multivariate regression analysis, the chance of PCD being the etiology of bronchiectasis increased with the presence of upper airway disease (chronic rhinosinusitis and/or nasal polyps; adjusted OR [aOR], 6.3; 95% CI, 3.3-11.9; *P* < .001), age < 53 years (aOR, 5.3; 95% CI, 2.7-10.4; *P* < .001), radiologic involvement of any middle and lower lobe (aOR, 3.7; 95% CI, 1.3-10.8; *P* = .016), duration of bronchiectasis > 15 years (aOR, 3.6; 95% CI, 1.9-6.9; *P* < .001), and a history of *Pseudomonas aeruginosa* isolation from respiratory specimen (aOR, 2.4; 95% CI, 1.3-4.5; *P* = .007).

**Interpretation:**

Within our nationally representative cohort, PCD was a common etiology of bronchiectasis. We identified few easy-to-assess phenotypic features, which may promote awareness for PCD among adults with bronchiectasis.

**Clinical Trial Registration:**

ClinicalTrials.gov; No.: NCT02574143; URL: www.clinicaltrials.gov

**Take-home Points**

**Study Question:** How common is primary ciliary dyskinesia (PCD) among adults with bronchiectasis, how do people with PCD differ clinically from people with other etiologies, and which clinical characteristics are independently associated with the diagnosis of PCD?

**Results:** Among the adult participants of our nationally representative bronchiectasis registry, PCD was a common etiology of bronchiectasis with distinct phenotype in 9% of patients. The presence of upper airway disease, younger age, radiologic predominance of middle and lower lobe bronchiectasis, long-standing duration of bronchiectasis, and a history of *Pseudomonas aeruginosa* isolation from respiratory specimen were independently associated with PCD.

**Interpretation:** Few easy-to-assess clinical variables may guide suspicion and justify referral to specific PCD diagnostics.

Bronchiectasis is a heterogeneous clinical syndrome with multiple underlying causes and associated conditions, and globally increasing prevalence.^1^, ^2^, ^3^ It is supported by the radiologic evidence of permanent bronchial dilatation and characterized by the presence of chronic cough, sputum production, and a history of pulmonary exacerbations and reduced health-related quality of life (QOL).^4^^,^^5^ Despite structured etiologic workup, even now its etiology remains undetermined in up to 38% of cases,^3^^,^^5^ which is referred to as idiopathic bronchiectasis.^4^^,^^6^, ^7^, ^8^

In contrast, primary ciliary dyskinesia (PCD) is a rare, genetic multisystem disorder with autosomal recessive, X-linked, or autosomal dominant inheritance caused by the malfunction of motile cilia.^9^ So far, pathogenic variants in > 50 genes are known to cause motile ciliopathies, resulting in remarkable genetic heterogeneity and considerable variability in the clinical phenotype.^9^^,^^10^ Historically, PCD with laterality defects (situs inversus) is referred to as Kartagener syndrome, which is defined as the triad of situs inversus, bronchiectasis, and chronic sinonasal disease.^11^ PCD is considered a specific etiology of bronchiectasis, with most people with PCD presenting with bronchiectasis in adulthood.^3^^,^^6^^,^^12^ Reported rates in which PCD was found to be the cause of bronchiectasis largely depend on variations between regions and countries, the expertise of respective centers, and the availability of appropriate diagnostics.^10^^,^^13^, ^14^, ^15^ Therefore, underdiagnosis of PCD is highly likely, as suggested by a UK genome sequencing study.^16^

Establishing the diagnosis of PCD in an adult with bronchiectasis is challenging for several reasons. First, the awareness for PCD is low in most nonspecialized care settings.^17^ Over the past years, several screening tools have been developed to facilitate the identification of patients with probable PCD and the discrimination from other etiologies of bronchiectasis.^18^, ^19^, ^20^, ^21^, ^22^, ^23^, ^24^ However, these tools largely rely on characteristic neonatal and early childhood history, limiting their use in adults, who often cannot recall the medical history of early life. Moreover, the clinical presentation of PCD varies with age. Although upper airway disease may dominate childhood, it often stands behind the symptoms of progressive lung disease with advancing age.^25^^,^^26^ Finally, there is no perfect single diagnostic test for PCD with sufficient sensitivity and specificity. Respective guidelines recommend sequential approaches with combinations of time-consuming and/or expensive tests requiring considerable resources and expertise. Therefore, only few specialized centers provide these laborious diagnostics, precluding the screening of all adults with bronchiectasis.^17^^,^^27^^,^^28^

However, the identification of PCD as the etiology of bronchiectasis in adults has important implications with respect to the management of extrapulmonary disease manifestations, genetic and fertility counseling, and/or the participation in disease-specific interventional trials.^29^, ^30^, ^31^ In this regard, we need a better understanding of the frequency of PCD among adult bronchiectasis and the clinical characteristics that help to distinguish people with PCD from those with other etiologies, to inquire PCD-specific clinical data and identify individuals for referral to diagnostic testing for PCD. Therefore, we aimed to determine the proportion, clinical phenotype, and variables independently associated with PCD among adults with bronchiectasis, using data from the Prospective German Non-CF-Bronchiectasis Registry (PROGNOSIS).

## Study Design and Methods

### Study Design

PROGNOSIS is an ongoing, prospective, multicenter observational cohort study consecutively enrolling adults with bronchiectasis from 38 sites across all levels of the German health care system since June 2015. The methodology of the registry has been described in detail elsewhere.^5^ Briefly, pseudonymized data are collected at baseline and annual follow-up visits (± 3 months) for up to 5 years using standardized electronic case report forms through an online database. In the present study, we used baseline data to assess the proportion of PCD as the etiology of bronchiectasis and clinical characteristics of people with PCD compared with those with other etiologies. In addition, we identified clinical variables that were predictive of PCD using a multivariate logistic regression model. Information on specific diagnostic tests were extracted from baseline and follow-up data up to a maximum of five follow-up visits or June 30, 2022, as applicable, to validate the diagnosis of PCD. For the purpose of our study, we ignored situs inversus (ie, we analyzed a patient with Kartagener syndrome as PCD) and pooled respective patients to enrich the cohort for evaluable observations. According to the noninterventional nature of our study, patients were managed locally without intervention from the registry team. PROGNOSIS was approved by the ethics committee of each participating center, with reference to the initial ethical approval of the institutional review board of Hannover Medical School (No. 6656/2015).

### Patient Population

Inclusion criteria were age ≥ 18 years, presence of CT scan-confirmed bronchiectasis affecting one or more lobes, a clinical history consistent with bronchiectasis, and prior written and informed consent. We excluded patients with bronchiectasis due to known cystic fibrosis and/or previous heart and lung transplantation.

### Data Collection

PROGNOSIS collects comprehensive clinical data beyond patient demographics and disease history (eg, disease severity and symptom burden, etiologic testing, lung function, radiology, microbiology, comorbidities, treatment). The impact of the disease and its treatment on daily life are assessed by the German translation of the Quality of Life Questionnaire-Bronchiectasis (QOL-B), version 3.1,^5^^,^^32^ whereas the radiologic manifestation of bronchiectasis is assessed by site investigators. Moreover, we assessed disease severity by calculating the multidimensional Bronchiectasis Severity Index, whenever possible.^33^ The etiology of bronchiectasis was determined locally and validated centrally based on the patients’ history and etiologic testing data (nasal nitric oxide, high-speed video microscopy, transmission electron microscopy [TEM], genetic testing, presence of Kartagener syndrome [situs inversus], or historical saccharin test), whenever possible. Notably, the German Bronchiectasis Registry PROGNOSIS was developed to capture neither the details of the characteristic history nor the specific disease manifestations of PCD.

### Statistical Analysis

We presented continuous data as mean ± SD or median (interquartile range) depending on their distribution and categorical data as total number and percentage. The Kolmogorov-Smirnov test was used to assess the distribution of continuous data. For the assessment of differences between groups, we used the Mann-Whitney *U* or the Kruskal-Wallis test for continuous data and the χ^2^ or Fisher exact test for categorical data, as appropriate. We applied the Bonferroni methods to correct for multiple comparisons. Missing values were infrequently observed (FEV_1_ in 64 patients, Medical Research Council Dyspnea Scale in 58 patients, number of pulmonary exacerbations in 26 patients, and BMI in two patients).^5^ Missing values were computed by the multiple imputation method based on the pattern of missingness and incorporating all clinically relevant and complete variables in the imputation model, with 10 iterations for each imputation cycle. We applied receiver operating characteristic curve analysis and calculated the Youden index to determine the optimal threshold for the continuous variable age for subsequent analysis. Clinical variables independently associated with the diagnosis of PCD were assessed by binary logistic regression analysis. Based on clinical relevance and previous literature, we entered all potential predictor or confounder variables of interest in the multivariate regression model simultaneously to estimate adjusted OR and 95% CI.^3^^,^^10^^,^^18^^,^^19^^,^^33^^,^^34^ However, we excluded treatments from multivariate analysis to avoid confounding bias by indication. *P* values and 95% CIs were calculated from Wald statistics and bootstrapping, respectively, with statistical significance set to *P* < .05. We used Hosmer-Lemeshow statistics to assess the goodness-of-fit of our model. There was no evidence of collinearity between variables. Analyses were conducted using SPSS, version 28.0 (IBM Corp). We generated figures using Excel 2016 (Microsoft Corp) and the freeware draw.io (JGraph Ltd).

## Results

### Patient Population

**Figure 1:**
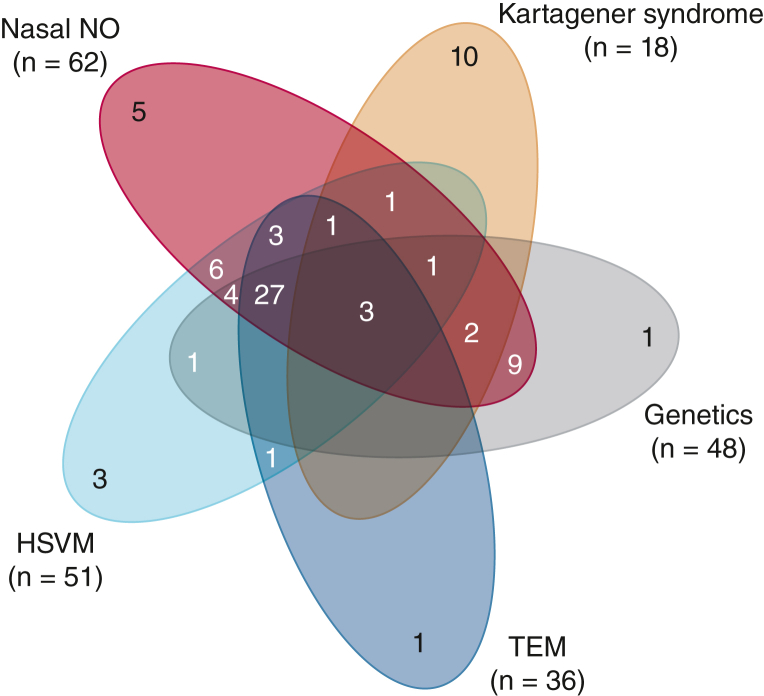
Venn diagram showing the diagnostic breakdown among 79 patients with primary ciliary dyskinesia, including 18 patients with Kartagener syndrome. HSVM = high-speed video microscopy; NO = nitric oxide; TEM = transmission electron microscopy.

Between July 2015 and March 2018, we enrolled 1,000 patients with bronchiectasis. Overall, 496 patients were recruited at university hospitals (50%), 290 at teaching hospitals (29%), and 214 at private respiratory practices (21%). In total, 275 patients were assessed for PCD with at least one diagnostic test (28%). Twelve sites recruited at least one patient with PCD, whereas only two sites contributed > 10 patients. The proportion of patients with PCD as the etiology of bronchiectasis per center ranged from 0% to 46% and was generally higher at university hospitals than teaching hospitals and private practices (15% vs 2%, each; *P* < .001) (e-Fig 1). Likewise, the proportion of patients tested for PCD showed large variation across centers (range, 0%-63%) and levels of health care (46% at university hospitals vs 11% at teaching hospitals and 9% at private practices; *P* < .001). This corresponded to a testing/etiology ratio of 3:1, 6:1, and 5:1 at university hospitals, teaching hospitals, and private practices, respectively (e-Fig 1). Idiopathic (n = 358; 36%), postinfective (n = 212; 21%), COPD (n = 149; 15%), and asthma (n = 111; 11%) were the most common etiologies followed by PCD as the fifth most prevalent etiology in 87 patients (9%), including 18 (21%) with Kartagener syndrome. Figure 1 shows a breakdown of the diagnostic workup of people with PCD. Altogether, 69 of 87 pwPCD (79%) received at least one recommended diagnostic test for PCD. Ten patients with Kartagener syndrome had no testing. Measurement of nasal nitric oxide was most frequently applied in 62 of 87 patients (71%), followed by high-speed video microscopy in 51 patients (59%), genetic testing in 48 patients (55%) and TEM in 38 patients (41%). The remaining 18 people with PCD had either Kartagener syndrome (n = 10) or historical saccharin test, which is no longer advocated as a diagnostic for PCD (n = 8). Notably, people with PCD without Kartagener syndrome were more often subject to recommended diagnostic testing than those with Kartagener syndrome (88% vs 44%, respectively), including confirmatory genetic testing and/or TEM (68% vs 39%, respectively) (e-Table 1, Fig 1).

### Clinical Phenotype of Adults With PCD

Table 1 shows the patients’ demographics. Patients with PCD were younger and more often had never smoked. Moreover, they had a lower mean BMI, a longer duration of bronchiectasis, more exacerbations, more often had a history of any prior bronchiectasis-associated hospitalization, and had more extensive sputum production, but less breathlessness. Compared with patients without PCD, they had radiologically more extensive bronchiectasis, which more frequently involved any middle and lower lobe. In contrast, we found no differences between groups regarding sex, airflow limitation, hospitalizations in the past 12 months, hemoptysis, and severity of bronchiectasis, as assessed by the Bronchiectasis Severity Index (Table 1).

**Table 1:** Baseline Characteristics of Adults With Bronchiectasis, Stratified by the Etiology of Bronchiectasis (PCD vs No PCD)

Characteristic	PCD (n = 87)	No PCD (n = 913)	*P* Value^a^
Age, y	42 ± 15	60 ± 14	< .001
Age categorized, y			< .001
18-29	19 (21.8)	47 (5.1)	
30-49	39 (44.8)	142 (15.6)	
50-69	22 (25.3)	420 (46.0)	
70-79	7 (8.0)	266 (29.1)	
≥ 80	0 (0)	38 (4.2)	
Female	57 (65.5)	536 (58.7)	.25
FEV_1_ % predicted	66.5 ± 23.1	69.6 ± 26.9	.24
FEV_1_ % predicted, categorized			.076
≥ 80	25 (28.7)	350 (38.3)	
50-79	41 (47.1)	319 (34.9)	
30-49	18 (20.7)	184 (20.2)	
< 30	3 (3.4)	60 (6.6)	
BMI, kg/m^2^	22.8 ± 3.7	24.2 ± 4.6	.001
BMI, kg/m^2^, categorized			.50
< 18.5	7 (8.1)	81 (8.9)	
18.5-30	75 (86.2)	747 (81.8)	
> 30	5 (5.8)	85 (9.3)	
Duration of bronchiectasis > 15 y	64 (73.6)	240 (26.3)	< .001
Duration of bronchiectasis, y			< .001
< 5	9 (10.3)	352 (38.6)	
5-10	7 (8.0)	153 (16.8)	
11-15	4 (4.6)	60 (6.6)	
16-20	12 (13.8)	53 (5.8)	
> 20	52 (59.8)	187 (20.5)	
Unknown	3 (3.4)	108 (11.8)	
Smoking status			< .001
Current tobacco use	3 (3.4)	62 (6.8)	
Previous tobacco use	13 (14.9)	358 (39.2)	
Never smoked	71 (81.6)	493 (54.0)	
MRC dyspnea scale, categorized			.010
1-3	81 (93.6)	755 (82.7)	
4-5	6 (6.4)	158 (17.3)	
Regular sputum production	82 (94.3)	698 (76.5)	< .001
Average daily sputum volume, mL	30 (10-50)	10 (0-25)	< .001
Average sputum volume, mL/d, categorized			< .001
0	7 (8.0)	304 (33.3)	
1-10	23 (26.4)	261 (28.6)	
11-20	12 (13.8)	116 (12.7)	
21-50	25 (28.7)	155 (17.0)	
51-100	14 (16.1)	53 (5.8)	
> 100	6 (6.9)	24 (2.6)	
Exacerbations in the past 12 mo	3 (1-4)	1 (0-3)	< .001
Exacerbations in the past 12 mo, categorized			< .001
0	17 (19.5)	287 (31.4)	
1-2	26 (29.9)	368 (40.3)	
≥ 3	44 (50.6)	258 (28.3)	
Hospitalizations in the past 12 mo	0 (0-1)	0 (0-1)	.93
Any hospitalization in the past 12 mo	30 (34.5)	357 (39.1)	.42
Any prior hospitalization due to bronchiectasis	65 (74.7)	542 (59.4)	.006
Significant hemoptysis (ever)	13 (14.9)	95 (10.4)	.20
BSI^b^	8 (4-11)	8 (5-12)	.064
BSI, categorized^b^			.038
Mild (0-4)	24 (29.3)	147 (22.5)	
Moderate (5-8)	38 (46.3)	398 (60.9)	
Severe (≥ 9)	20 (24.4)	109 (16.7)	
Radiologic extent			< .001
< 3 lobes affected	15 (17.2)	356 (39.0)	
≥ 3 lobes affected	52 (59.8)	411 (45.0)	
Cystic bronchiectasis	20 (23.0)	146 (16.0)	
Involvement of any middle and lower lobe	76 (87.4)	502 (55.0)	< .001

Data are presented as No. (%), mean ± SD, median (interquartile range), or as otherwise indicated. BSI = Bronchiectasis Severity Index; MRC = Medical Research Council; PCD = primary ciliary dyskinesia.

a Differences between groups were assessed by the χ^2^ and Fisher exact test, as appropriate (categorical data), and the Mann-Whitney *U* test, according to the distribution of data (continuous data).

b We could not calculate the BSI in all patients due to missing data on repeat sputum microbiology defining chronic infection (n = 736).

As an integral part of their disease manifestation, upper airway disease (chronic rhinosinusitis and/or nasal polyps) was much more common in people with PCD (78% vs 28%; *P* < .001). In contrast, cardiovascular comorbidities, COPD, malignancy, renal insufficiency, and diabetes were less frequent (Table 2). We observed no differences between groups regarding the frequency of asthma, osteoporosis, self-reported gastroesophageal reflux, liver cirrhosis, and mental disorders (Table 2).

**Table 2:** Comorbidities of Adults With Bronchiectasis, Stratified by the Etiology of Bronchiectasis (PCD vs No PCD)

Condition	PCD (n = 87)	No PCD (n = 913)	*P* Value^a^
Chronic rhinosinusitis	66 (75.0)	227 (24.9)	< .001
Nasal polyps	45 (51.1)	110 (12.1)	< .001
Cardiovascular	14 (15.9)	369 (40.5)	< .001
COPD	4 (4.5)	303 (33.3)	< .001
Asthma	22 (25.0)	271 (29.7)	.46
Malignancy	2 (2.3)	115 (12.6)	.002
Renal insufficiency	0 (0.0)	73 (8.0)	.002
Diabetes	2 (2.3)	86 (9.4)	.027
Osteoporosis	5 (5.7)	99 (10.9)	.20
Gastroesophageal reflux (self-reported)	18 (20.5)	170 (18.6)	.67
Liver cirrhosis	0 (0.0)	11 (1.2)	.61
Anxiety disorder	1 (1.1)	38 (4.2)	.25
Depression	9 (10.2)	90 (9.9)	.85

Data are presented as No. (%) or as otherwise indicated. PCD = primary ciliary dyskinesia.

a Differences between groups were assessed by χ^2^ and Fisher exact test, as applicable.

Overall, *Pseudomonas aeruginosa*, *Staphylococcus aureus*, *Haemophilus influenzae*, *Aspergillus fumigatus*, and nontuberculous mycobacteria were the most common pathogens among patients with evaluable respiratory cultures at baseline and/or within the previous 12 months from baseline in 33%, 16%, 14%, 10%, and 9% of patients, respectively. Although *P aeruginosa*, *H influenzae*, and *A fumigatus* were more frequently cultured in people with PCD, we observed no differences between groups for *S aureus* and nontuberculous mycobacteria (Table 3). Moreover, people with PCD more often had an all-time history of a positive airway culture for *P aeruginosa* compared with patients with other etiologies of bronchiectasis (66% vs 32%, respectively; *P* < .001).

**Table 3:** Microbiology of Respiratory Specimens Among Adults With Bronchiectasis, Stratified by the Etiology of Bronchiectasis (PCD vs No PCD)

Pathogen	PCD (n = 83)	No PCD (n = 673)	*P* Value^a^
*Pseudomonas aeruginosa* cultured (ever)	57 (65.5)	295 (32.3)	< .001
*P aeruginosa*^b^	44 (53.0)	205 (30.5)	< .001
*Staphylococcus aureus*^b^	16 (19.3)	108 (16.0)	.44
*Haemophilus influenzae*^b^	21 (25.3)	82 (12.2)	.002
*Aspergillus fumigatus*^b^	14 (16.9)	65 (9.7)	.006
*Nontuberculous mycobacteria*^b^,^c^	2 (2.9)	46 (9.5)	.070

Data are presented as No. (%) or as otherwise indicated. PCD = primary ciliary dyskinesia.

a Differences between groups were assessed by χ^2^ and Fisher exact test, as appropriate.

b Microbiology at baseline or in the previous 12 mo (n = 756).

c Refers to patients who were tested for mycobacteria at baseline or in the previous 12 mo (n = 555).

### Treatment of Bronchiectasis and QOL

**Figure 2:**
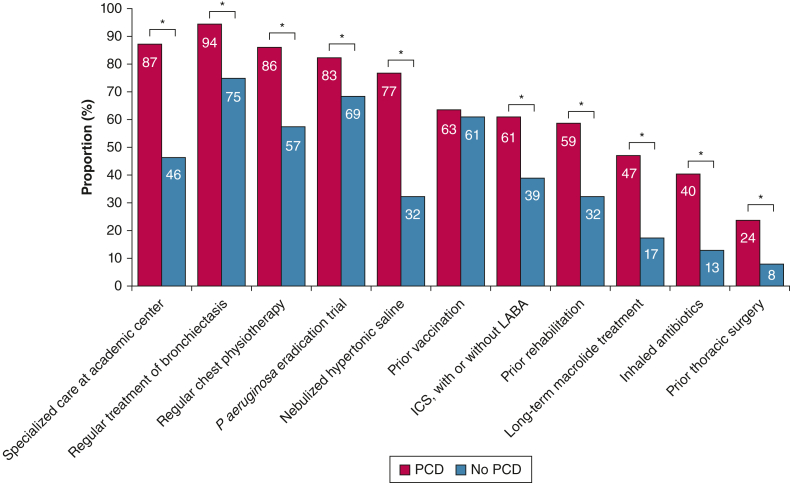
Proportion of applied treatment modalities, stratified by the etiology of bronchiectasis (PCD vs no PCD; N = 1,000). Differences between groups were assessed by the χ^2^ or Fisher exact test for categorical data, as appropriate. ICS = inhaled corticosteroid; LABA = long-acting beta agonist; PCD = primary ciliary dyskinesia. ∗*P* < .001; ∗∗*P* < .05.

**Figure 3:**
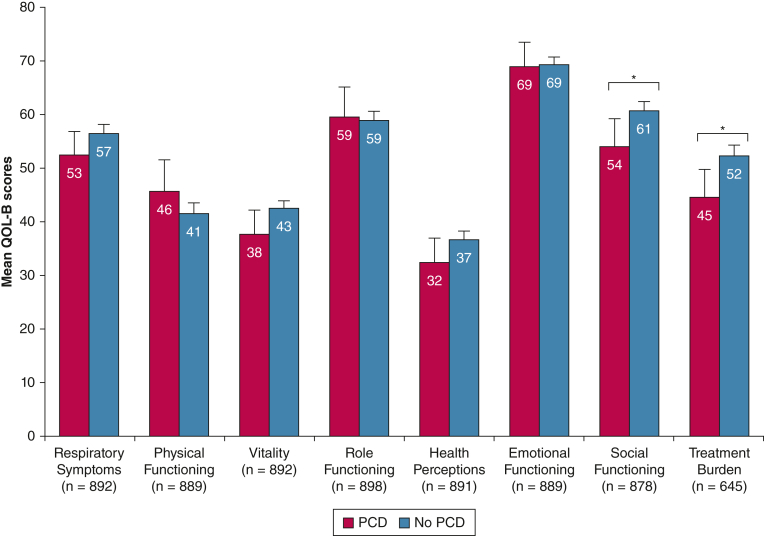
Column chart showing mean QOL-B scores, stratified by the etiology of bronchiectasis (PCD vs no PCD). Error bars represent 95% CI. Differences between groups were assessed by the Mann-Whitney U test. Note that the number of patients with evaluable QOL-B scores varies with each QoL-B score. ∗*P* < .05. PCD = primary ciliary dyskinesia; QOL-B = Quality of Life Questionnaire-Bronchiectasis.

Patients with PCD more frequently attended specialized care facilities at tertiary care centers than those without PCD (87% vs 46%, respectively; *P* < .001), with regular treatment of bronchiectasis applied in most people with PCD (e-Table 2, Fig 2). Apparently, people with PCD more frequently used nonpharmacologic and pharmacologic therapies than patients with other etiologies, including regular chest physiotherapy, prior rehabilitation, prior thoracic surgery, and various drug treatments. However, we observed no difference regarding prior vaccinations (e-Table 2, Fig 2). Accordingly, people with PCD had worse QOL-B scores for the Treatment Burden and Social Functioning scale, whereas we found no further differences between groups for any other QOL-B scale (e-Table 3, Fig 3).

### Predictive Clinical Variables of PCD Among Adults With Bronchiectasis

**Figure 4:**
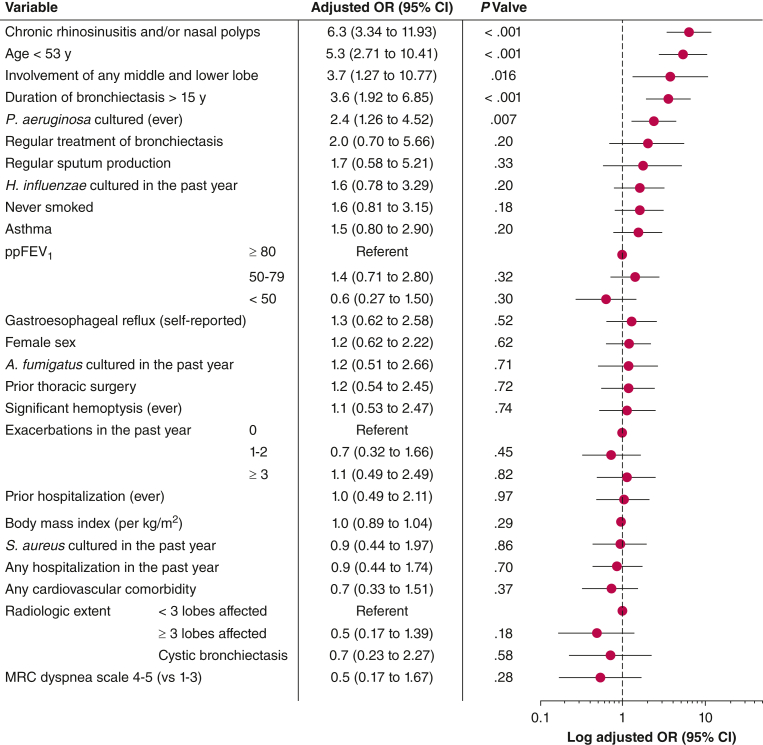
Clinical variables independently associated with primary ciliary dyskinesia as the etiology of bronchiectasis in adults (N = 1,000): multivariate logistic regression analysis and respective forest plots of adjusted ORs and 95% CIs. ppFEV_1_ = percent predicted FEV_1_; MRC = Medical Research Council.

We dichotomized age according to receiver operating characteristic curve analysis and subsequent calculation of the Youden index for application in our multivariate logistic regression model. Age < 53 years indicated the best discriminatory power for PCD as the etiology of bronchiectasis (e-Fig 2, e-Table 4). Using a comprehensive regression model, we observed that the presence of upper airway disease (chronic rhinosinusitis and/or nasal polyps) increased the chance of PCD being the etiology of bronchiectasis about sixfold (Fig 4). Likewise, age < 53 years, radiologic involvement of any middle and lower lobe, duration of bronchiectasis > 15 years, and a history of at least one positive airway culture of *P aeruginosa* increased this likelihood about fivefold, fourfold, fourfold, and twofold, respectively. No further independent associations were identified (Fig 4).

### Discussion

To our knowledge, our study provides a first estimate of the proportion of PCD as the etiology of bronchiectasis derived from a nationally representative cohort of adults with bronchiectasis in Germany. Moreover, we demonstrated that people with PCD had a distinct clinical phenotype compared with other etiologies of bronchiectasis, which we used to identify easy-to-assess clinical features that may help to raise awareness of PCD.

Remarkably, PCD was the fifth most frequent underlying cause of bronchiectasis in this cohort in 9% of patients. This frequency is much higher than previously reported in adults with bronchiectasis. Both the US Bronchiectasis Research Registry and the European Multicentre Bronchiectasis Audit and Research Collaboration (EMBARC) found rates of 3% each,^3^^,^^35^ whereas the respective Australian and Spanish registries reported PCD as the etiology of adult bronchiectasis in 4% each.^36^^,^^37^ A UK whole genome sequencing substudy of the 100,000 Genomes Project may provide an explanation for our finding.^16^ This study found pathogenic or likely pathogenic variants in motile ciliopathy genes in 12% of individuals with bronchiectasis and so far unknown PCD, strongly supporting underdiagnosis of PCD in bronchiectasis. In addition, data reported from a UK national audit showed that < 2% of 4,898 audited patients with bronchiectasis were tested for PCD compared with rates of overall 28% and even 46% at university hospitals in our study. In this regard, the observed frequency of PCD among adults with bronchiectasis of at least 9% in our study could even be higher with more widespread diagnostic coverage. In the past few years, we observed an increased uptake of genetic testing for orphan diseases in Germany and a comparatively high rate of confirmative genetic testing in up to 61% of people with PCD without Kartagener syndrome among this study cohort.

Furthermore, our findings provide a detailed clinical characterization of people with PCD and bronchiectasis. Based on this particular phenotype, we identified five easy to assess clinical features that characterized people with PCD among adults with bronchiectasis. We observed that people with PCD had a higher chance of being younger and having upper airway disease, a radiologic involvement of any middle and lower lobe, a longer duration of bronchiectasis, and a history of *P aeruginosa* isolation. These findings are consistent with the previous literature. A descriptive analysis of the US Bronchiectasis Research Registry included a comparable number of 79 people with PCD at the same mean age of 42 years, with an earlier diagnosis of bronchiectasis and a similarly high rate of *P aeruginosa* (64% vs 66% in our study).^38^ Compared with these results, our study provides a more detailed picture on the clinical phenotype, including a substantiated multivariate analysis of variables independently associated with the diagnosis of PCD.

Due to the striking lack of awareness for PCD, several clinical and radiologic prediction tools have been published over the last years.^18^, ^19^, ^20^, ^21^, ^22^^,^^24^ The most commonly used scores are the Primary Ciliary Dyskinesia Rule^18^ and the North American Criteria Defined Clinical Features,^19^ which were validated in external cohorts without obvious differences in performance.^21^^,^^22^ Both the Primary Ciliary Dyskinesia Rule and the North American Criteria Defined Clinical Features were derived from predominantly pediatric cohorts with median ages of 9 and 8 years, respectively. Accordingly, both scores strongly rely on early life history and PCD-specific clinical features (eg, chronic ear or hearing symptoms). In contrast, our findings were based on readily available routine data from adult care and did not include information about the early childhood period, thus avoiding the risk of recall bias. To our knowledge, so far only one French study constructed a diagnostic prediction tool from a cohort of adults with bronchiectasis.^24^ In summary, Schlemmer et al^24^ made similar observations, with age < 15 years at symptom onset, chronic ear, nose, and throat disorders, and isolation of *P aeruginosa* being predictive of PCD among adults with bronchiectasis. However, contrary to our study, this single-center study included only 12 people with PCD (out of 158 patients with bronchiectasis) and primarily aimed to identify patients at high risk of having either cystic fibrosis or PCD.^24^ Interestingly, we observed a comparatively low frequency of people with PCD with situs inversus (Kartagener syndrome) of 21% among the present study cohort. The reasons for this finding remain elusive, but may relate to the earlier diagnosis of Kartagener syndrome with subsequent referral to dedicated and mostly pediatric PCD centers that did not contribute to our registry or the predominance of specific genotypes associated with normal left-right body asymmetry.^9^

Notably, the lifelong and more extensive treatment of people with PCD appeared to translate into reduced QOL regarding treatment burden and social functioning, thus highlighting the need for more effective and easier to deliver treatments in this particular patient group.^31^^,^^39^, ^40^, ^41^

Our study has strengths and limitations. The PROGNOSIS registry contributed in-depth real-life data from a large number of individuals with bronchiectasis across all levels of the German health care system with a widespread geographic distribution. This ensured national representativeness and allowed us to conduct a comprehensive analysis with a rigorous statistical approach. In addition, a comparatively large proportion of people with PCD included in our analysis had confirmative diagnostics for PCD (ie, genetics and/or TEM in up to 68% of people with PCD without Kartagener syndrome), allowing validation of PCD in most patients, and only few patients were diagnosed based on a single diagnostic test result along with clinical features and history. However, the key limitation of our study is that we did not design PROGNOSIS as a PCD registry. Therefore, some specific clinical features of PCD are lacking (eg, neonatal history, chronic ear and hearing symptoms, congenital heart defects, infertility/subfertility, family history/consanguinity), whereas the granularity on others is low. Unfortunately, we were unable to validate the outcomes of diagnostic testing in detail (eg, detected ultrastructural defects, genetic variants), therefore depending on the site investigators' judgment. Our study included only adult patients with PCD and bronchiectasis. In this regard, we acknowledge that the ultimate goal should be to diagnose PCD as early in life as possible to prevent structural lung damage. However, given the large number of undiagnosed adults with PCD and bronchiectasis, our results have important implications and confirm and expand existing knowledge on the clinical phenotype and features independently associated with PCD among adults with bronchiectasis. Deliberately, we refrained from formally constructing a prediction tool because we are unable to exclude some extent of confirmation bias, even though three times more patients had been tested for PCD than finally diagnosed (275 vs 87 patients). We propose to be rather inclusive regarding referral for PCD-specific diagnostics in patients with suggestive clinical features, in particular keeping the considerable genetic and clinical heterogeneity of people with PCD in mind, which we unfortunately were unable to account for. The generalizability of our findings could have been biased by hierarchical clustering by site (ie, the fact that most people with PCD were recruited at few of the 38 participating sites). However, these large-volume university hospitals have expertise in managing adults with bronchiectasis and specifically PCD and nationwide referral structures and recruited people with PCD, who resided in at least 14 of the 16 German federal states, thus supporting national representativeness of our findings. Finally, it should be mentioned that age is a major component of the Bronchiectasis Severity Index and thus may not be suitable for the assessment of disease severity in people with PCD.

## Interpretation

We showed that PCD is a common cause of bronchiectasis among adults in Germany. People with PCD showed a distinct clinical phenotype and reduced QOL, in particular relating to burdensome treatment and psychosocial aspects. Younger adults with bronchiectasis, concomitant upper airway disease, predominant middle and lower lobe involvement, a long-standing history of disease, or a history of *P aeruginosa* isolation should be questioned about the specific clinical features of PCD and considered for referral to further diagnostic testing for PCD, in particular with specific counseling for family planning and future, genotype-directed treatment options in mind.

## Funding/Support

The German bronchiectasis registry PROGNOSIS is financially supported by 10.13039/100015739Bayer Vital, Grifols SA, Insmed Inc, InfectoPharm, the German Ministry of Research and Education (BMBF) via the 10.13039/501100010564German Center for Lung Research (DZL) and the German Center for Infection Research (DZIF), and the European bronchiectasis registry EMBARC via the 10.13039/501100010767Innovative Medicines Initiative (IMI) and EFPIA companies under the European Commission funded project, iABC [Grant 115721]. CAPNETZ STIFTUNG supported the PROGNOSIS registry concerning building up the successful study network, the multicenter database (and adjunct biobank), and data management to ensure consistently high quality of data. The authors participate in the BEAT-PCD Clinical Research Collaboration, supported by the ERS.

## Financial/Nonfinancial Disclosures

The authors have reported to *CHEST* the following: I. P. reports grants from COFONI-2FF4 and COFONI-6LZF23 by the Ministry of Science and Culture of Lower Saxony paid to her institution and personal lecture fees from AstraZeneca and Boehringer Ingelheim. S. S. reports fees for clinical trial participation from Celtaxsys, Corbus, Galapagos, Insmed, Proteostasis, and Vertex Pharmaceuticals paid to his institution; and personal fees for consulting and lectures from Boehringer Ingelheim, Insmed, and Vertex Pharmaceuticals. S. P. A. reports personal consulting fees from AstraZeneca, Boehringer Ingelheim, Daiichi-Sankyo, and GlaxoSmithKline. A. G. reports personal fees for consulting, advisory board participation, and lectures from Boehringer Ingelheim and GlaxoSmithKline. A. S. reports personal fees for consulting and lectures from Ethris, Insmed, Spirovant, and Translate Bio; and is involved in the European Respiratory Society (ERS) Clinical Research Collaborations AMR-Lung, BEAT-PCD, and EMBARC. S. W. reports fees for clinical trial participation from Insmed paid to her institution and personal honoraria from Vertex Pharmaceuticals. P. M. reports fees for clinical trial participation from Boehringer Ingelheim and Insmed paid to his institution; reports personal fees for lectures from AstraZeneca, MAÄF eV, ResMed, and streamedup! GmbH; reports travel support from the German Society for Internal Medicine (DGIM), CSL Behring, and Insmed; and is honorary co-chair of the German Bronchiectasis Registry PROGNOSIS. J. R. reports grants from the German Center for Lung Research (DZL), the German Center for Infection Research (DZIF), the Federal Ministry of Education and Research (BMBF), the Federal Ministry of Health (BMG), Novartis, and Insmed paid to her institution; reports personal fees for consulting or advisory board participation and honoraria for lectures from AstraZeneca, Brahms GmbH, ERS, Grifols, Insmed, MedUpdate, MSD, Pfizer, Shionogi, and streamedup! GmbH; and is honorary co-chair of the German Bronchiectasis Registry PROGNOSIS and chair of the Respiratory Infections and Tuberculosis Assembly of the German Respiratory Society (DGP). F. C. R. reports grants from the German Center for Lung Research (DZL), the German Center for Infection Research (DZIF), IMI (EU/EFPIA), and the Innovative Medicines Initiative (IMI) and EFPIA companies under the European Commission funded project, iABC [Grant 115721], Novartis, and Insmed Germany paid to his institution; reports personal fees for consulting or advisory board participation and honoraria for lectures from Parion, Grifols, Zambon, Insmed, Helmholtz-Zentrum für Infektionsforschung, i!DE Werbeagentur GmbH, Interkongress GmbH, streamedup! GmbH, AstraZeneca, Insmed, Shionogi, and Grifols; reports travel support from the German Kartagener Syndrome and Primary Ciliary Dyskinesia patient advocacy group, which he serves as the unpaid co-speaker of its medical advisory board; and is honorary co-chair of the German Bronchiectasis Registry PROGNOSIS, a member of the steering committee of the European Bronchiectasis Registry EMBARC, and a member of the Protocol Review Committee of the PCD-Clinical Trials Network. None declared (R. E., U. S., B. O. S.).

## Supplementary Data

e-Online Data
