# Supplementary material for: Primary Ciliary Dyskinesia in Adult Bronchiectasis: Data from the German Bronchiectasis Registry PROGNOSIS
Source: Chest. 2024 Jun 15;166(5):938–50. doi: 10.1016/j.chest.2024.05.023 (PMC11562653; doi:10.1016/j.chest.2024.05.023)
Supplement: e-Online Data [file mmc1.docx]

Primary Ciliary Dyskinesia in Adult Bronchiectasis – Data from the German Bronchiectasis Registry PROGNOSIS

Raphael Ewen, MD; Isabell Pink, MD; Sivagurunathan Sutharsan, MD; Sven P. Aries, MD; Achim Grünewaldt, MD; Amelia Shoemark, PhD; Urte Sommerwerck, MD; Ben O. Staar, MD; Sabine Wege, MD; Pontus Mertsch, MD;* Jessica Rademacher, MD;* Felix C. Ringshausen, MD*, and the PROGNOSIS Study group

– Supplemental material –


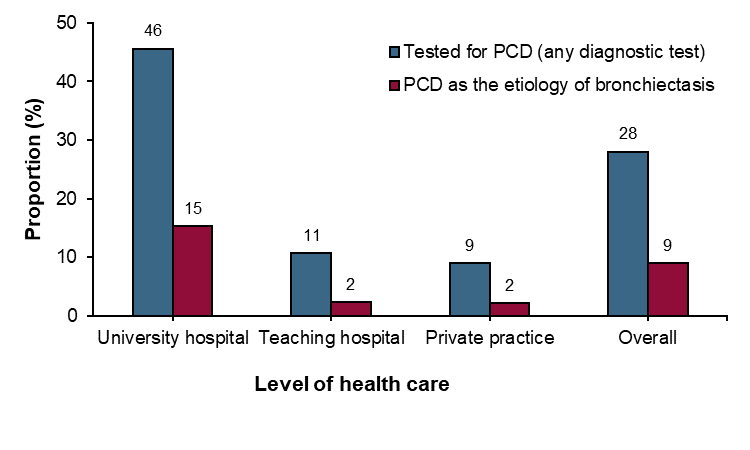


**e-Figure 1** *– Testing for PCD and PCD as the etiology of bronchiectasis, overall and stratified by level of health care*

**e-Table 1.** Breakdown of PCD diagnostics

|  | | **PCD/KS** | **PCD** | **KS** |
| --- | --- | --- | --- | --- |
| N (%) | | 87 (100) | 69 (79.3) | 18 (20.7) |
| Genetic testing | | 48 (55.2) | 42 (60.9) | 6 (33.3) |
| Nasal NO | | 62 (71.3) | 54 (78.3) | 8 (44.4) |
| TEM | | 36 (41.4) | 32 (46.4) | 4 (22.2) |
| HSVM | | 51 (58.6) | 45 (65.2) | 6 (33.3) |
| Genetic testing and/or TEM | | 54 (62.1) | 47 (68.1) | 7 (38.9) |
| No. of diagnostic tests | ≥ 1 | 69 (79.3) | 61 (88.4) | 8 (44.4) |
|  | ≥ 2 | 59 (67.8) | 51 (73.9) | 8 (44.4) |
|  | ≥ 3 | 39 (44.8) | 34 (49.3) | 5 (27.8) |
|  | ≥ 4 | 30 (34.5) | 27 (39.1) | 3 (16.7) |

HSVM = high-speed video microscopy; KS = Kartagener syndrome; NO = nitric oxide; PCD = primary ciliary dyskinesia; TEM = transmission electron microscopy

**e-Table 2.** Treatments

| **Treatment** | **PCD**  (n = 87) | **No PCD**  (n = 913) | ***P* Value**^a^ |
| --- | --- | --- | --- |
| Specialized care at academic center | 76 (87.4) | 420 (46.0) | <0.001 |
| Regular treatment of bronchiectasis | 82 (94.3) | 684 (74.9) | <0.001 |
| Regular chest physiotherapy | 75 (86.2) | 520 (57.0) | <0.001 |
| Prior thoracic surgery | 21 (23.9) | 69 (7.6) | <0.001 |
| Prior rehabilitation | 51 (58.6) | 296 (32.4) | <0.001 |
| Prior vaccination | 55 (63.2) | 559 (61.2) | 0.82 |
| Inhaled antibiotics | 35 (40.2) | 117 (12.8) | <0.001 |
| Long-term macrolide treatment | 41 (47.1) | 157 (17.2) | <0.001 |
| ICS, with or without LABA | 53 (60.9) | 356 (39.0) | <0.001 |
| Nebulized hypertonic saline | 67 (77.0) | 295 (32.3) | <0.001 |
| *Pseudomonas aeruginosa* eradication trial^b^ | 47 (82.5) | 202 (68.5) | 0.038 |

Data are presented as *n* (%). ICS = inhaled corticosteroids; LABA = long-acting β2 adrenergic receptor agonists; PCD = primary ciliary dyskinesia.

^a^Differences between groups were assessed by Chi-square and Fisher’s exact test, as applicable.

^b^Referring to the 352 subjects with *Pseudomonas aeruginosa* ever cultured.

**e-Table 3.** QOL-B scores stratified by the etiology of bronchiectasis (PCD vs No PCD)^a^

|  | **Mean (SD) QOL-B scores** | |  |
| --- | --- | --- | --- |
| **QOL-B scale** | **PCD**  (n = 78) | **No PCD**  (n = 826) | ***P* Value^b^** |
| Respiratory Symptoms (n = 892) | 52.6 (18.2) | 56.5 (21.3) | 0.12 |
| Physical Functioning (n = 889) | 45.7 (25.7) | 41.4 (30.2) | 0.12 |
| Vitality (n = 892) | 37.7 (19.5) | 42.5 (21.5) | 0.062 |
| Role Functioning (n = 898) | 59.4 (24.4) | 58.8 (27.7) | 0.94 |
| Health Perceptions (n = 891) | 32.4 (19.3) | 36.7 (22.9) | 0.15 |
| Emotional Functioning (n = 889) | 68.9 (20.1) | 69.2 (22.0) | 0.77 |
| Social Functioning (n = 878) | 53.8 (23.7) | 60.5 (27.1) | 0.014 |
| Treatment Burden (n = 645)^c^ | 44.6 (21.7) | 52.1 (25.4) | 0.020 |

PCD = primary ciliary dyskinesia; QOL-B = Quality of Life Questionnaire-Bronchiectasis; SD = standard deviation.

^a^Referring to subjects in whom at least one baseline QOL-B scale was evaluable (n = 904).

^b^Differences between groups were assessed by the Mann-Whitney U test.

^c^Patients not receiving bronchiectasis treatment were instructed to skip the Treatment Burden scale.


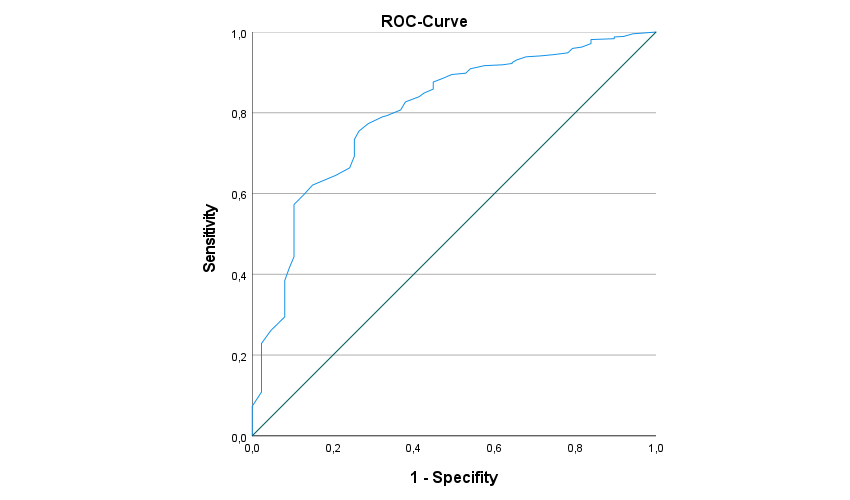


**e-Figure 2** *– Receiver operating characteristics curve for age at baseline as a predictive variable of PCD (AUC 0.802, 95% CI 0.754 – 0.850; P < .001)*

**e-Table 4.** Youden index analysis for assessment of the best predictive threshold for age at baseline

| **Positive, if greater or equal to** | **Sensitivity** | **1 - Specifity** | **Specifity** | **Youden index** |
| --- | --- | --- | --- | --- |
| 17 | 1 | 1 | 0 | 0 |
| 18.5 | 0.996 | 0.943 | 0.057 | 0.053 |
| 19.5 | 0.989 | 0.92 | 0.08 | 0.069 |
| 20.5 | 0.988 | 0.897 | 0.103 | 0.091 |
| 21.5 | 0.986 | 0.897 | 0.103 | 0.089 |
| 22.5 | 0.984 | 0.897 | 0.103 | 0.087 |
| 24 | 0.981 | 0.839 | 0.161 | 0.142 |
| 25.5 | 0.978 | 0.839 | 0.161 | 0.139 |
| 26.5 | 0.972 | 0.839 | 0.161 | 0.133 |
| 27.5 | 0.963 | 0.816 | 0.184 | 0.147 |
| 28.5 | 0.959 | 0.793 | 0.207 | 0.166 |
| 29.5 | 0.949 | 0.782 | 0.218 | 0.167 |
| 30.5 | 0.944 | 0.747 | 0.253 | 0.197 |
| 31.5 | 0.941 | 0.713 | 0.287 | 0.228 |
| 32.5 | 0.939 | 0.678 | 0.322 | 0.261 |
| 33.5 | 0.931 | 0.655 | 0.345 | 0.276 |
| 34.5 | 0.924 | 0.644 | 0.356 | 0.28 |
| 35.5 | 0.922 | 0.644 | 0.356 | 0.278 |
| 36.5 | 0.919 | 0.621 | 0.379 | 0.298 |
| 37.5 | 0.917 | 0.575 | 0.425 | 0.342 |
| 38.5 | 0.909 | 0.54 | 0.46 | 0.369 |
| 39.5 | 0.898 | 0.529 | 0.471 | 0.369 |
| 40.5 | 0.895 | 0.494 | 0.506 | 0.401 |
| 41.5 | 0.885 | 0.471 | 0.529 | 0.414 |
| 42.5 | 0.876 | 0.448 | 0.552 | 0.428 |
| 43.5 | 0.866 | 0.448 | 0.552 | 0.418 |
| 44.5 | 0.859 | 0.448 | 0.552 | 0.411 |
| 45.5 | 0.849 | 0.425 | 0.575 | 0.424 |
| 46.5 | 0.84 | 0.414 | 0.586 | 0.426 |
| 47.5 | 0.827 | 0.379 | 0.621 | 0.448 |
| 48.5 | 0.807 | 0.368 | 0.632 | 0.439 |
| 49.5 | 0.793 | 0.333 | 0.667 | 0.46 |
| 50.5 | 0.79 | 0.322 | 0.678 | 0.468 |
| 51.5 | 0.773 | 0.287 | 0.713 | 0.486 |
| **52.5** | **0.755** | **0.264** | **0.736** | **0.491** |
| 53.5 | 0.734 | 0.253 | 0.747 | 0.481 |
| 54.5 | 0.711 | 0.253 | 0.747 | 0.458 |
| 55.5 | 0.692 | 0.253 | 0.747 | 0.439 |
| 56.5 | 0.664 | 0.241 | 0.759 | 0.423 |
| 57.5 | 0.645 | 0.207 | 0.793 | 0.438 |
| 58.5 | 0.621 | 0.149 | 0.851 | 0.472 |
| 59.5 | 0.596 | 0.126 | 0.874 | 0.47 |
| 60.5 | 0.573 | 0.103 | 0.897 | 0.47 |
| 61.5 | 0.545 | 0.103 | 0.897 | 0.442 |
| 62.5 | 0.53 | 0.103 | 0.897 | 0.427 |
| 63.5 | 0.494 | 0.103 | 0.897 | 0.391 |
| 64.5 | 0.463 | 0.103 | 0.897 | 0.36 |
| 65.5 | 0.444 | 0.103 | 0.897 | 0.341 |
| 66.5 | 0.416 | 0.092 | 0.908 | 0.324 |
| 67.5 | 0.384 | 0.08 | 0.92 | 0.304 |
| 68.5 | 0.359 | 0.08 | 0.92 | 0.279 |
| 69.5 | 0.333 | 0.08 | 0.92 | 0.253 |
| 70.5 | 0.318 | 0.08 | 0.92 | 0.238 |
| 71.5 | 0.295 | 0.08 | 0.92 | 0.215 |
| 72.5 | 0.261 | 0.046 | 0.954 | 0.215 |
| 73.5 | 0.229 | 0.023 | 0.977 | 0.206 |
| 74.5 | 0.191 | 0.023 | 0.977 | 0.168 |
| 75.5 | 0.149 | 0.023 | 0.977 | 0.126 |
| 76.5 | 0.108 | 0.023 | 0.977 | 0.085 |
| 77.5 | 0.073 | 0 | 1 | 0.073 |
| 78.5 | 0.059 | 0 | 1 | 0.059 |
| 79.5 | 0.042 | 0 | 1 | 0.042 |
| 80.5 | 0.034 | 0 | 1 | 0.034 |
| 81.5 | 0.023 | 0 | 1 | 0.023 |
| 82.5 | 0.02 | 0 | 1 | 0.02 |
| 83.5 | 0.018 | 0 | 1 | 0.018 |
| 84.5 | 0.013 | 0 | 1 | 0.013 |
| 86 | 0.007 | 0 | 1 | 0.007 |
| 87.5 | 0.004 | 0 | 1 | 0.004 |
| 89 | 0.003 | 0 | 1 | 0.003 |
| 90.5 | 0.002 | 0 | 1 | 0.002 |
| 92 | 0.001 | 0 | 1 | 0.001 |
| 94 | 0 | 0 | 1 | 0 |

Youden Index *J* the best predictive threshold for age at baseline indicated in **bold**.
